# *In-situ* reconstruction of CoBO_x_ enables formation of Co for synthesis of benzylamine through reductive amination

**DOI:** 10.3389/fchem.2022.1104844

**Published:** 2023-01-04

**Authors:** Mingkai Zhang, Sai Zhang, Yuanyuan Ma

**Affiliations:** ^1^ Key Laboratory of Special Functional and Smart Polymer Materials of Ministry of Industry and Information Technology, School of Chemistry and Chemical Engineering, Northwestern Polytechnical University, Xi’an, China; ^2^ Frontier Institute of Science and Technology, Xi’an Jiaotong University, Xi’an, China

**Keywords:** heterogeneous catalysis, reductive amination, primary amine, in-situ reconstruction, cobalt

## Abstract

Cobalt (Co) as a substitute of noble-metal catalysts shows high catalytic capability for production of the widely used primary amines through the reductive amination. However, the synthesis of Co catalysts usually involves the introduction of organic compounds and the high-temperature pyrolysis, which is complicated and difficult for large-scale applications. Herein, we demonstrated a facile and efficient strategy for the preparation of Co catalysts through the *in situ* reconstruction of cobalt borate (CoBO_x_) during the reductive amination, delivering a high catalytic activity for production of benzylamine from benzaldehyde and ammonia. Initially, CoBO_x_ was transformed into Co(OH)_2_ through the interaction with ammonia and subsequently reduced to Co nanoparticles by H_2_ under the reaction environments. The *in situ* generated Co catalysts exhibited a satisfactory activity and selectivity to the target product, which overmatched the commonly used Co/C, Pt or Raney Ni catalysts. We anticipate that such an *in situ* reconstruction of CoBO_x_ by reactants during the reaction could provide a new approach for the design and optimization of catalysts to produce primary amines.

## 1 Introduction

Primary amines are essential raw materials and intermediates in fine chemical industry, playing significant roles in the synthesis of pharmaceuticals, biomolecules, advanced polymers, and agrochemicals (Kim et al., 2013; Cabrero-Antonino et al., 2019; Afanasyev et al., 2020; Irrgang and Kempe, 2020; Murugesan, et al., 2020). Various strategies have been investigated to synthesize primary amines, including reductive amination (Ball et al., 2018; Murugesan et al., 2019; Sukhorukov, 2020), aryl halides amination (Schranck and Tlili, 2018; Wang et al., 2022), nitriles hydrogenation (Garduño and García, 2020; Lévay and Hegedűs, 2018; Chen et al., 2016), olefins hydroamination (Miller et al., 2019; Sengupta et al., 2020), etc. Owing to the application of economical ammonia as well as the accessible reaction engineering, the reductive amination with usage of ammonia as the nitrogen resource represents one of the most cost-effective methods to manufacture primary amines. However, this process usually suffers from the selectivity challenge due to the side reactions including over hydrogenation of imines and reduction of carbonyl compounds to the corresponding alcohols (Chandra et al., 2018; Gallardo-Donaire et al., 2018). Thus, to design the appropriate catalyst to achieve selective production of primary amines through the reductive amination is highly desired.

Both traditional homogeneous metal complexes and heterogeneous catalysts have been successfully applied to produce primary amines through reductive amination, in which heterogeneous catalysts are more applicable in industrial field with the advantages of good durability, easy recycling and high potential for scale-up. Previously, noble metal-based catalysts (Pt, Pd, Ru, Rh, etc.) have exhibited satisfactory selectivity for target product (Dong et al., 2015; Nakamura et al., 2015; Chatterjee et al., 2016; Komanoya et al., 2017; Liang et al., 2017; Guo et al., 2019; Dong et al., 2020; Jv et al., 2020; Qi et al., 2021). Nevertheless, the high price of noble metals increases the production cost and limits the practical application. Consequently, the development of the non-noble metal-based heterogeneous catalysts is highly expected.

On account of the relatively high abundance and low price, Co has been employed as a reasonable non-noble metal-based catalysts in reductive amination among different substitutes for noble metals. As shown in Table 1, several heterogeneous Co-based catalysts were successfully reported (Jagadeesh et al., 2017; Liu et al., 2019; Yuan et al., 2019; Senthamarai et al., 2020; Ma et al., 2022), including Co-DABCO-TPA@C-800, Co@NC-800, Co/mCN-900, etc. With Co nanoparticles as the active sites, these catalysts delivered satisfactory reactivity and realized the selective synthesis of benzylamine. Generally, the synthesis of the reported Co nanocatalysts was achieved through the thermal pyrolysis of various organometallic cobalt complexes. Although such synthetic method has been proved efficacious to prepare Co-based catalysts, the complicated operating procedures and high-temperature pyrolysis constrain its further application. Thus, an alternative method with the simplified procedure and mild condition is highly demanded.

**TABLE 1:** Comparison on different Co-based catalysts in reductive amination.

Number	Catalyst	Reaction conditions	Yield of benzylamine (%)	Ref
1	Co@NC-700	110°C, NH_3_·H_2_O, 2 MPa H_2_	92	Liu et al., 2019
2	Co@NC-ligand-800	120°C, 0.5–0.7 MPa NH_3_	74	Ma et al., 2022
3	Co-DABCO-TPA@C-800	120°C, 0.5–0.7 MPa NH_3_, 4 MPa H_2_	87	Jagadeesh et al. (2017)
4	Co@NC-800	130°C, NH_3_·H_2_O, 1 MPa H_2_	97	Yuan et al., 2019
5	Co-salen complexes	120°C, 0.5 MPa NH_3_, 4.5 MPa H_2_	89	Senthamarai et al. (2020)

Herein, we reported a facile method to prepare Co catalysts through the *in situ* reconstruction of CoBO_x_ under the reductive amination environment, which delivered a high catalytic activity for the production of benzylamine between benzaldehyde and ammonia. In this method, the original CoBO_x_ reacted with ammonia to yield surface Co(OH)_2_ initially, which was then reduced by hydrogen (H_2_) to form Co nanoparticles during the reductive amination. In comparison with the previously reported strategies to prepare Co catalysts, such reconstructed Co catalysts realized the *in situ* formation of Co active sites and avoided the introduction of organic complexes as well as the high-temperature pyrolysis, in accordance with the requirements for green chemistry. The *in situ* synthesized Co catalysts delivered high capability for the selective conversion of benzaldehyde and ammonia towards benzylamine (> 95%). This reconstruction synthetic method may provide a new approach for the design of non-noble metal-based heterogeneous catalysts for reductive amination.

## 2 Materials and methods

### 2.1 Synthesis of catalysts

#### 2.1.1 Synthesis of CoBO_x_

Based on a previous work (Chen et al., 2016), the CoBO_x_ nanosheets were synthesized through a facile and scalable wet chemistry method. Typically, 3 mmol of Co(NO_3_)_2_·6H_2_O and 7.5 mmol of NaBH_4_ were dissolved in 285 ml and 15 ml distilled water, respectively. Then, the freshly prepared aqueous NaBH_4_ solution was added into the Co(NO_3_)_2_ solution and the mixture was placed under the stirring of 800 rpm at room temperature for 1 h. After 2 h of aging, the solids were centrifuged off and washed by distilled water and ethanol alternatively for three times. Finally, the collected CoBO_x_ was dried at 60°C for 12 h in a vacuum oven and then stored for future use.

#### 2.1.2 Synthesis of C and SiO_2_ supports

C supports were synthesized from the nitric acid treatment of the commercial carbon black. The details could be found in a previous report (Zhang et al., 2022). To synthesize SiO_2_ supports, 300 mg of the commercial SiO_2_ was well dispersed in 50 ml of ethanol, then 100 μl of 3-(Trimethoxysilyl)-1-propanamine was added into the suspension. Next, the mixture was heated under 90°C for 4 h and washed by ethanol for three times. Finally, the prepared SiO_2_ was dried at 60°C for 24 h and stored in a vacuum oven.

#### 2.1.3 Synthesis of Pt-based catalysts

The Pt/C catalysts were synthesized through an impregnation method. Firstly, 300 mg of the nitric acid-treated carbon black was well dispersed in 30 ml of distilled water by ultrasonication. Then, 0.75 ml of 2 mg ml^−1^ of Na_2_PtCl_6_·6H_2_O was added and the suspension was placed under the stirring of 800 rpm for 1 h at room temperature. Afterwards, the mixture was heated at 80°C until the complete evaporation of the solution and the collected solid was treated under H_2_/Ar atmosphere at 300°C for 2 h. Finally, the Pt/C catalysts were dried in vacuum at 60°C for 12 h. The Pt/SiO_2_ catalysts were prepared through the similar procedure.

#### 2.1.4 Synthesis of Co/C

To synthesize Co/C catalysts, 300 mg of the nitric acid-treated carbon black was well dispersed in 30 ml of distilled water by ultrasonication. Then, 3 ml of 5 mg ml^−1^ Co(NO_3_)_2_·6H_2_O solution was added and the suspension was placed under the stirring of 800 rpm for 1 h at room temperature. Afterwards, the mixture was heated at 80°C until the complete evaporation of the solution and the collected solid was treated under H_2_/Ar atmosphere at 300°C for 2 h with a heating rate of 5°C/min. Finally, the Co/C catalysts were dried and stored in a vacuum oven.

#### 2.1.5 Synthesis of Co(OH)_2_ and Co_3_O_4_

To synthesize Co(OH)_2_, 1.16 g of Co(NO_3_)_2_·6H_2_O and 19.2 g of NaOH were dissolved in 10 ml and 70 ml of distilled water, respectively. After 1 h of rigorous stirring, the two solutions were mixed and then transferred to a 100 ml of stainless autoclave. After 100°C of hydrothermal for 24 h, the solids were centrifuged off and washed by distilled water and ethanol alternatively for three times. Finally, the collected Co(OH)_2_ was dried at 60°C in a vacuum oven for 12 h and then stored for future use. For the synthesis of Co_3_O_4_, 300 mg of the as-synthesized CoBO_x_ was calcinated at 500°C under air for 3 h, then the solids were collected and stored after cooling to room temperature.

### 2.2 Characterizations

Transmission electron microscopy (TEM) measurements were conducted on Hitachi HT-7700 with the accelerating voltage of 120 kV. High resolution transmission electron microscopy (HRTEM) was performed on a JEOL JEM-F200 microscope with an accelerating voltage of 200 kV. X-ray diffraction (XRD) patterns were acquired from a Rigaku Powder X-ray diffractometer with the Cu Kα radiation, and X-ray photoelectron spectra (XPS) were obtained on a Thermo Electron Model K-Alpha with Al Kα as the excitation sources.

### 2.3 Catalytic reaction

The reductive amination of benzaldehyde and aqueous ammonia was carried out in a 30 ml of stainless autoclave. Initially, the CoBO_x_ catalysts, benzaldehyde, ammonia and isopropanol with the desired amounts were mixed in the autoclave. Next, the stainless autoclave was pressurized with 2 MPa of H_2_, and the reaction temperature was raised to 80°C with a heating rate of 5°C/min. Finally, the reaction was processed under the stirring (600 rpm) at 80°C. When the reaction finished, the reaction mixture was centrifugated off and diluted with ethyl acetate. After the dehydration by anhydrous magnesium sulfate, the reaction products were analyzed by gas chromatography. The conversion of benzaldehyde (*X*) and the product selectivity (*S*_*i*_) were calculated by the following equations:

$$X\left(\%\right)=\frac{{\left[Benzaldehyde\right]}_{0}-{\left[Benzaldehyde\right]}_{t}}{{\left[Benzaldehyde\right]}_{0}}\times 100$$

$$S_{i}\left(\%\right)=\frac{{\left[Product\right]}_{i}\times N_{i}}{{\left[Benzaldehyde\right]}_{0}-{\left[Benzaldehyde\right]}_{t}}\times 100$$

Where [*Benzaldehyde*]_*0*_ and [*Benzaldehyde*]_*t*_ were the initial concentration and the residual concentration of benzaldehyde, respectively. [*Product*]_*i*_ was the concentration of the product *i* in the residual mixture and *N*_*i*_ was the stoichiometric coefficient for product *i* with reference to benzaldehyde.

## 3 Results and discussion

**FIGURE 1:**
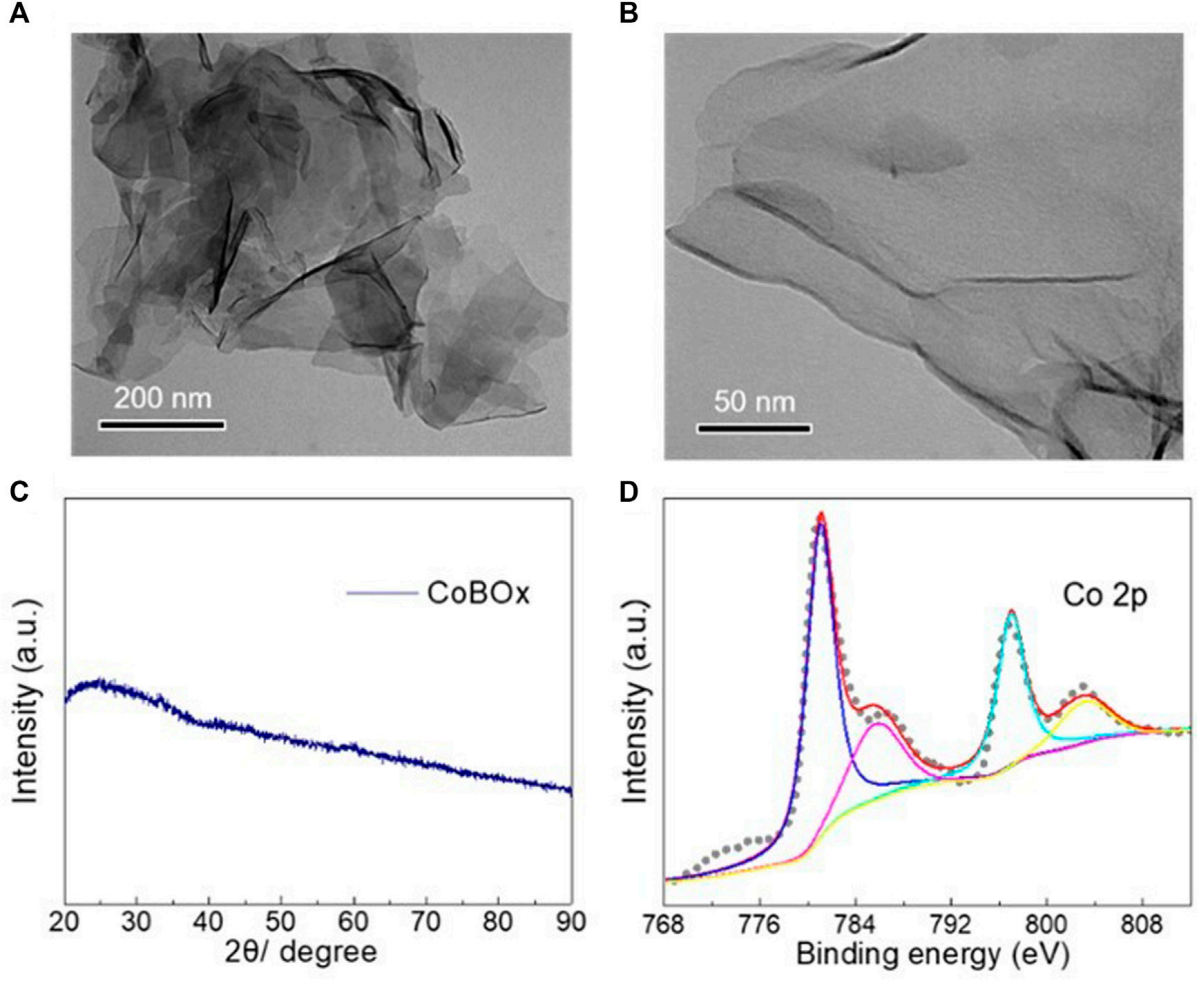
Characterizations of the fresh CoBO_x_ catalysts. **(A,B)** TEM images of the CoBO_x_ nanosheets with different magnification times. **(C)** XRD pattern and **(D)** XPS spectrum of Co 2p of the CoBO_x_ nanosheets.

The morphology of the as-synthesized CoBO_x_ catalysts was examined by the transmission electron microscopy. As shown in Figures 1A, B, the as-synthesized CoBO_x_ nanomaterials exhibited a nanosheet morphology with the smooth surface. No XRD characteristic peaks were observed for the CoBO_x_ nanosheets, indicating their amorphous feature (Figure 1C). Afterwards, the valence state of Co element in CoBO_x_ catalysts was analyzed by XPS. As shown in Figure 1D, Co^2+^ primarily existed on the surface of CoBO_x_, indicated by the characteristic peaks at 780.9 eV and the presence of a satellite peak. Meanwhile, there was no apparent peak at approximate 778.0 eV, further confirming the absence of Co^0^ in the as-synthesized nanosheets.

**FIGURE 2:**
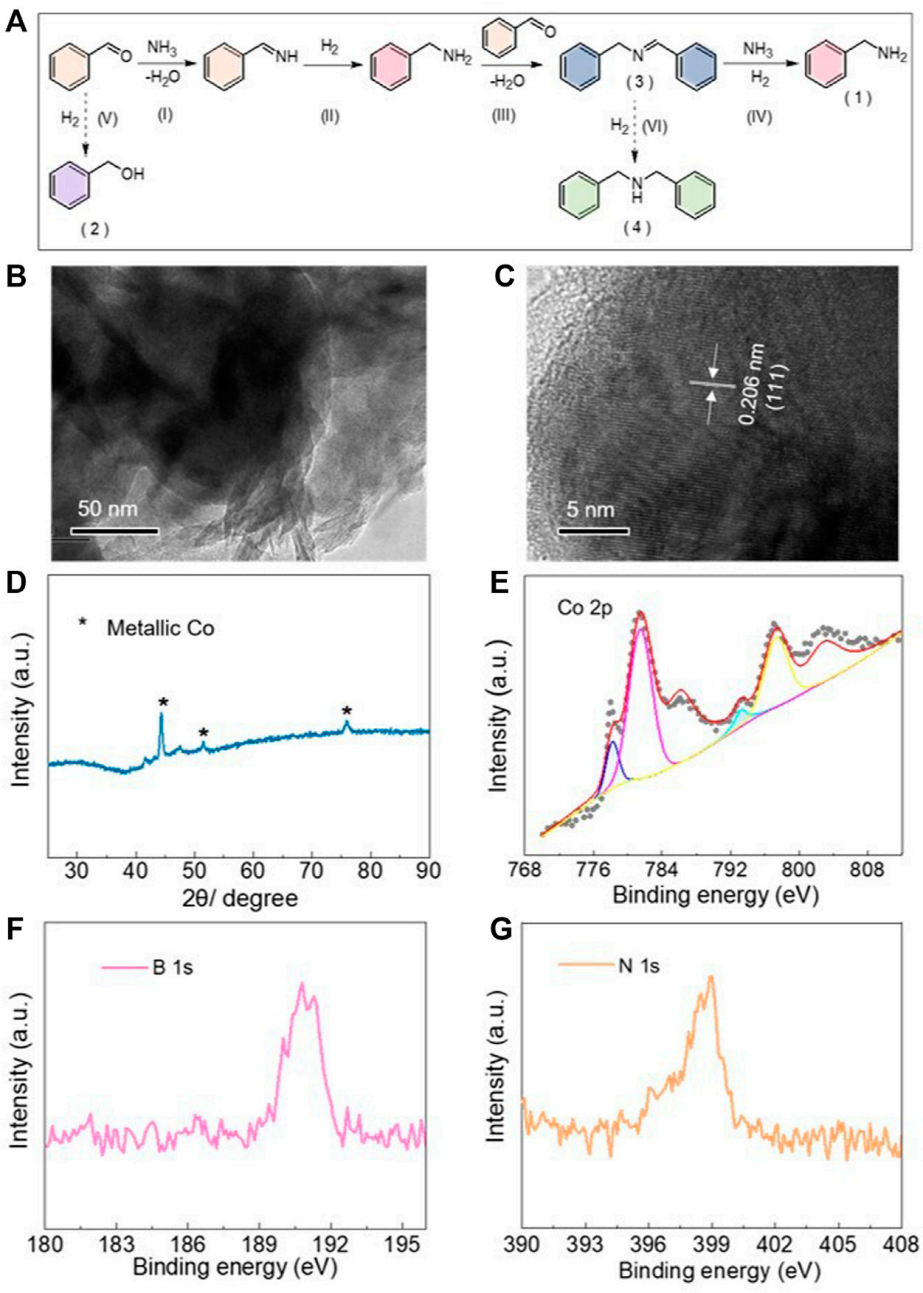
**(A)** Possible reaction pathway for the reductive amination between benzaldehyde and ammonia. **(B)** TEM and **(C)** HRTEM images of the reconstructed CoBO_x_. **(D)** XRD pattern and **(E)** XPS spectrum of Co 2p of the treated CoBO_x_. **(F)** XPS spectrum of B 1s and **(G)** N 1s of the treated CoBO_x_.

The reductive amination between benzaldehyde and aqueous ammonia (25 *wt*%–28 *wt*%) to produce benzylamine was applied to explore the *in situ* reconstruction of CoBO_x_ as well as the catalytic performance. Generally, the following steps are included in the reductive amination of benzaldehyde and ammonia into benzylamine (Figure 2A): (I) the condensation between benzaldehyde and ammonia to generate benzylideneimine; (II) the reduction of benzylideneimine to benzylamine; (III) the condensation between the produced benzylamine and benzaldehyde to intermediate *N*-benzylidenebenzylamine; and (IV) the following reductive amination of *N*-benzylidenebenzylamine to target product benzylamine (product 1). Nevertheless, the side reactions of (V) the hydrogenation of benzaldehyde into benzyl alcohol (product 2) and (VI) the over-hydrogenation of *N*-benzylidenebenzylamine (product 3) into dibenzylamine (product 4) decrease the selectivity of the target product, limiting the practical application of this process.

Considering the cation exchange between Co^2+^ and NH_4_^+^ ions of ammonia and the existence of hydrogen in this reaction system, Co nanoparticles could be reconstructed on the surface of CoBO_x_ in the presence of ammonia with a high concentration and H_2_. To further examine this possibility, the CoBO_x_ nanosheets were mixed with ammonia, benzaldehyde, and isopropanol (IPA) under 80°C and 2 MPa of H_2_ for 15 h. Afterwards, the treated CoBO_x_ was centrifuged off and characterized in comparison with the freshly prepared CoBO_x_. Different from the as-synthesized CoBO_x_ with the light grey color and fluffy accumulation, the treated CoBO_x_ was in black color with a needle-like appearance, which displayed a strong magnetic response (Supplementary Figure S1). Considering the strong magnetism of the metallic Co, the as-synthesized CoBO_x_ might be *in situ* transformed into Co nanoparticles in the presence of ammonia and H_2_.

Afterwards, the treated CoBO_x_ was analyzed by various characterizations. Derived from the dark field TEM and HRTEM (Figures 2B, C), the morphology of the treated CoBO_x_ was totally distinguished from the as-synthesized CoBO_x_. The original nanosheet structure was obviously destructed. As shown in Figure 2C, the measured lattice fringe spacing of 0.206 nm was observed, which was consistent with that of Co (111) crystal plane, suggesting the existence of Co nanoparticles in the treated CoBO_x_. In addition, the formation of the Co nanoparticles was further revealed from its XRD pattern. As shown in Figure 2D, the XRD pattern of the treated CoBO_x_ was in accordance with that of metallic Co (PDF 15–0806), in which a sharp peak could be found around 44.5°, and other small peaks was approximate in 51.6° and 76.1°, respectively. Besides, small peaks at around 41.7° and 47.8° could be attributed to the formation of Co_2_B. To further prove the existence of the Co^0^, XPS measurements were also employed. As shown in XPS spectrum of Co 2p (Figure 2E), the characteristic peak at 778.1 eV and the peak at 780.9 eV as well as satellite peak indicated the co-existence of Co^0^ and Co^2+^ in the reconstructed catalysts. The derived fraction of Co^0^ was only 14.7%, which could be attributed to the residual CoBO_x_ as well as the oxidation of cobalt upon the exposure of catalysts in air during the measurements. In addition, apparent peaks observed on B 1s and N 1s spectrum revealed the presence of B and N along with the generation of Co nanoparticles (Figures 2F, G). The XPS results were in accordance with that of TEM and XRD tests, suggesting the successful *in situ* generation of Co nanoparticles. As expected, the original CoBO_x_ nanosheets could react with ammonia under H_2_ atmosphere during the reductive amination of benzaldehyde and ammonia.

**FIGURE 3:**
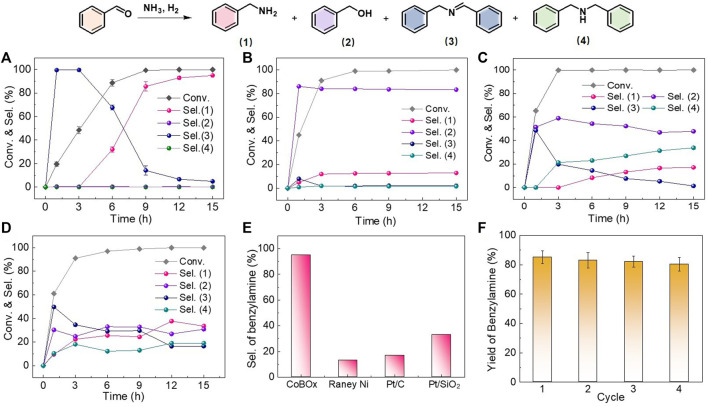
Catalytic performance of **(A)** CoBO_x_, **(B)** Raney Ni, **(C)** Pt/C and **(D)** Pt/SiO_2_ catalysts. **(E)** Comparison on the selectivity of benzylamine at the end of the reaction. **(F)** Cycling test of CoBO_x_ catalysts. Reaction conditions: 0.5 mmol benzaldehyde, 80°C, 2 MPa H_2_, 4 ml IPA, 20 mg CoBO_x_, or 20 mg 0.5 *wt*% Pt/C, 20 mg 0.5 *wt*% Pt/SiO_2_, or 5 mg Raney Ni catalysts. The error bars in **(A,F)** were calculated based on three trials.

Then, the catalytic performance of the reconstructed Co nanoparticles was performed for the reductive amination between benzaldehyde and ammonia. After the optimization of solvent (Supplementary Figure S2), the reaction was conducted in isopropanol under 2 MPa of H_2_ and 80°C. Meanwhile, the catalytic performance of the Raney Ni, Pt/C, and Pt/SiO_2_ as the controlled catalysts was also examined. When the reconstructed CoBO_x_ was used as the catalysts (Figure 3A), benzaldehyde was converted to intermediate *N*-benzylidenebenzylamine at the initial 3 h (Step I-III), and further hydrogenated to the target benzylamine as reaction proceeded (Step IV). Finally, the conversion of benzaldehyde reached to > 99% with a selectivity of benzylamine of 95.2% at 15 h. Except for the intermediate *N*-benzylidenebenzylamine and the target product benzylamine, by products of benzyl alcohol and dibenzylamine were not detected, indicating the high reactivity and chemoselectivity of the reconstructed CoBO_x_ catalysts. In contrast, the main product appeared to be benzyl alcohol on Raney Ni. The selectivity of benzylamine was only 12.1% after the 15 h, indicating that the Step V was predominant on Raney Ni (Figure 3B). On Pt-based catalysts, by products of benzyl alcohol and dibenzylamine were both detected, indicating the occurrence of the Step V and Step VI. The selectivity of benzylamine on Pt/C and Pt/SiO_2_ were 17.1% and 33.6%, respectively (Figures 3C, D). According to the experimental results, the Co nanoparticles generated from the *in situ* reconstruction of CoBO_x_ could effectively inhibit the side reactions of benzaldehyde direct hydrogenation and *N*-benzylidenebenzylamine over hydrogenation, avoiding the production of by-products and exhibiting a superior selectivity towards benzylamine in the reductive amination between benzaldehyde and ammonia. As shown in Figure 3E, the selectivity of benzylamine on CoBO_x_ was 7.9, 5.6, and 2.8 times of that on Raney Ni, Pt/C and Pt/SiO_2_, respectively.

Afterwards, the stability of the *in situ* formed Co nanoparticles was examined by cycling test. Due to the strong magnetism, the used catalysts could be easily separated from the reaction mixture by a magnet. Without other treatments, the collected catalysts were applied for the next cycle on the identical reaction conditions. As shown in Figure 3F, the reaction time was controlled as 9 h and the catalysts could be reused at least four cycles with the yields of benzylamine maintained over 80%, suggesting the good catalytic stability of the *in situ* formed Co nanoparticles. Meanwhile, the reaction mixture was analyzed by inductively coupled plasma optical emission spectrometer (ICP-OES), and Co was not detected. The slight reduction of the yield of benzylamine might be resulted from the catalyst loss during the cycling test.

Based on the characterizations and the experimental results, it could be confirmed that CoBO_x_ could react with ammonia and H_2_ under the reductive amination environments, resulting in the formation of Co nanoparticles through the *in situ* reconstruction. Moreover, the Co nanoparticles efficiently suppressed the side reactions and realized the selective production of benzylamine. In order to clarify the active sites on the reconstructed CoBO_x_, various Co-based catalysts were prepared. As shown in Supplementary Table S1, Co_3_O_4_ and Co(OH)_2_ were synthesized by the calcination of CoBO_x_ and the hydrothermal of Co(NO_3_)_2_·6H_2_O, respectively (Supplementary Figures S3, S4). The yield of benzylamine was below 1% when catalyzed by either Co_3_O_4_ or Co(OH)_2_. Thus, the metallic Co should play a significant role in the production of benzylamine. Afterwards, the Co/C catalysts were prepared and employed in this reaction, presenting an obvious lower yield of benzylamine (40.0%) compared with the *in situ* generated Co nanoparticles. As a result, the high selectivity of re-constructed CoBO_x_ was not only attributed to the formation of metallic Co, but also the *in situ* catalytic environment.

**FIGURE 4:**
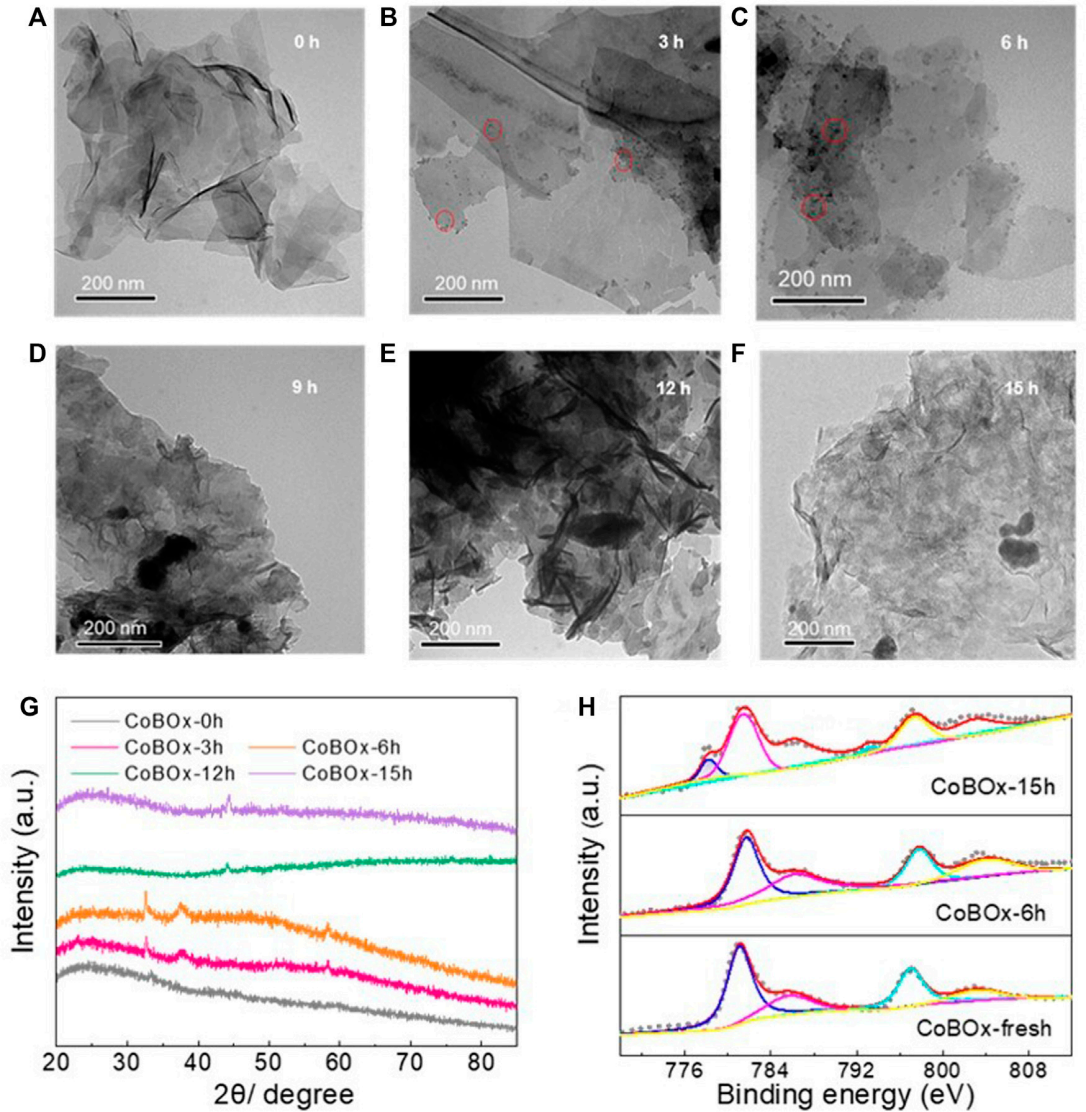
TEM images of **(A)** CoBO_x_-fresh, **(B)** CoBO_x_-3 h, **(C)** CoBO_x_-6 h, **(D)** CoBO_x_-9 h, **(E)** CoBO_x_-12 h, **(F)** CoBO_x_-15 h **(G)** XRD pattern and **(H)** XPS spectrum of Co 2p of different CoBO_x_ catalysts.

Afterwards, the reconstruction pathway of CoBO_x_ was explored. The reconstruction of CoBO_x_ should be a dynamic process, including the reduction of Co^2+^ to Co^0^ and the crystallization of metallic Co. To further investigate the dynamic change of CoBO_x_ during the reductive amination between benzaldehyde and ammonia, the catalysts were collected after the 3 h, 6 h, 9 h, 12 h, and 15 h reaction, respectively, which are named by CoBO_x_-3 h, CoBO_x_-6 h, CoBO_x_-9 h, CoBO_x_-12 h, CoBO_x_-15 h. After the thoroughly washing and vacuum drying, these catalysts were analyzed. According to TEM images, it could be observed that nanoparticles were formed on the surface of CoBO_x_ during the reaction and they aggregated and grew up as reaction proceeded (Figures 4B, C). To further demonstrate the reconstruction of CoBO_x_, XRD and XPS tests were performed on the various CoBO_x_ catalysts. According to Figure 4G, the peaks at 32.6°, 38.1°, and 58.2° were observed on CoBO_x_-3 h and CoBO_x_-6 h, which was in consistent with the characteristic peaks of Co(OH)_2_ (PDF no. 51–1731), indicating the formation of Co(OH)_2_ from CoBO_x_ during the first 6 h of the reaction. As the reaction continued, the characteristic peaks of Co(OH)_2_ disappeared, and the typical peaks of metallic Co were observed, indicating the reduction of Co(OH)_2_ to Co nanoparticles. Moreover, the XPS analysis exhibited the similar phenomenon, in which Co^0^ peaks couldn’t be found on the original CoBO_x_ and CoBO_x_-6 h, but existed in CoBO_x_-15 h (Figure 4H).

Integrating the results of TEM, XRD, and XPS, the *in situ* reconstruction of CoBO_x_ might be summarized into the following steps: 1) Given the high concentration of ammonia (∼4.5 mmol/ml) in the reaction system, NH_4_^+^ and OH^−^ could be easily formed through ionization and separately reacted with CoBO_x_ (Eq. 1). Consequently, Co^2+^ in CoBO_x_ could be substituted by NH_4_^+^ and then interacted with OH^−^, resulting in the formation of Co(OH)_2_ (Eqs 2, 3). 2) Under H_2_ atmosphere, Co(OH)_2_ was finally reduced to Co nanoparticles (Eq. 4). The chemical formulas of CoBO_x_ reconstruction process are shown below:

$${NH}_{3}+H_{2}O\;↔\;{NH}_{4}^{+}+{OH}^{-}$$ (1)

$${CoBO}_{x}+{NH}_{4}^{+}\;\to \;({NH}_{4}{{)}_{2}BO}_{x}+{Co}^{2+}$$ (2)

$${Co}^{2+}+{OH}^{-}\;\to \;{Co\left(OH\right)}_{2}$$ (3)

$${Co\left(OH\right)}_{2}+H_{2}\;\to \;Co+H_{2}O$$ (4)

**SCHEME 1:**
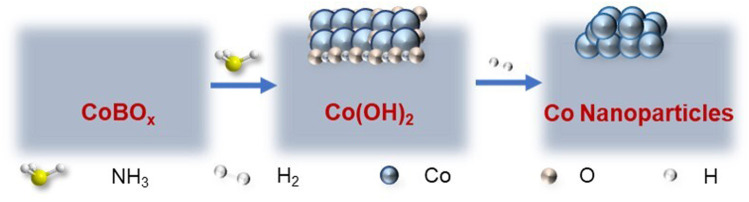
Formation of Co through *in situ* reconstruction of CoBO_x_.

As discussed above, the *in situ* reconstruction of CoBO_x_ follows the order of CoBO_x_→Co(OH)_2_→Co nanoparticles, with which benzaldehyde is selectively transformed to the target product benzylamine. The whole reconstruction process is demonstrated in Scheme 1. According to Scheme 1, ammonia and H_2_ play significant roles in the *in situ* reconstruction of CoBO_x_. To further investigate the influences of ammonia volume and H_2_ pressure, the CoBO_x_ nanosheets were treated under different volume of ammonia and pressure of H_2_. As shown in Supplementary Table S2, only when the volume of ammonia > 2 ml and the pressure of H_2_ > 2 MPa could the CoBO_x_ be *in situ* reconstructed. Thus, adequate ammonia volume and H_2_ pressure are necessary for the *in situ* reconstruction of CoBO_x_. Besides, NaOH was also applied to replace ammonia, it turned out that Co nanoparticles were not formed, further confirming the indispensability of ammonia.

## 4 Conclusion

In this work, we reported the formation of Co nanoparticles through the *in situ* reconstruction of CoBO_x_ during the reductive amination of benzaldehyde and ammonia, which delivered a high catalytic performance for the synthesis of benzylamine from benzaldehyde and ammonia. In the presence of ammonia and H_2_, the cation exchange between Co^2+^ and NH_4_^+^ leads to the formation of Co(OH)_2_ on CoBO_x_, which was subsequently reduced to Co nanoparticles. The *in situ* generated Co nanoparticles could suppress the side reactions and realize the selective reductive amination to give primary amine. The selectivity of benzylamine was 95.2% catalyzed by the reconstructed CoBO_x_, which was obvious higher than Raney Ni, Pt/C or Pt/SiO_2_ catalysts. Such an *in situ* reconstruction strategy provided a new approach for the synthesis of the highly performed metallic Co for production of primary amines.

## Data Availability

The original contributions presented in the study are included in the article/Supplementary Material, further inquiries can be directed to the corresponding authors.
