# Supplementary material for: In-situ reconstruction of CoBOx enables formation of Co for synthesis of benzylamine through reductive amination
Source: Front Chem. 2023 Jan 4;10:1104844. doi: 10.3389/fchem.2022.1104844 (PMC9845621; doi:10.3389/fchem.2022.1104844)
Supplement: Supplementary file 1 [file DataSheet1.docx]

Supplementary Material

# Supplementary Figures and Tables

## Supplementary Figures


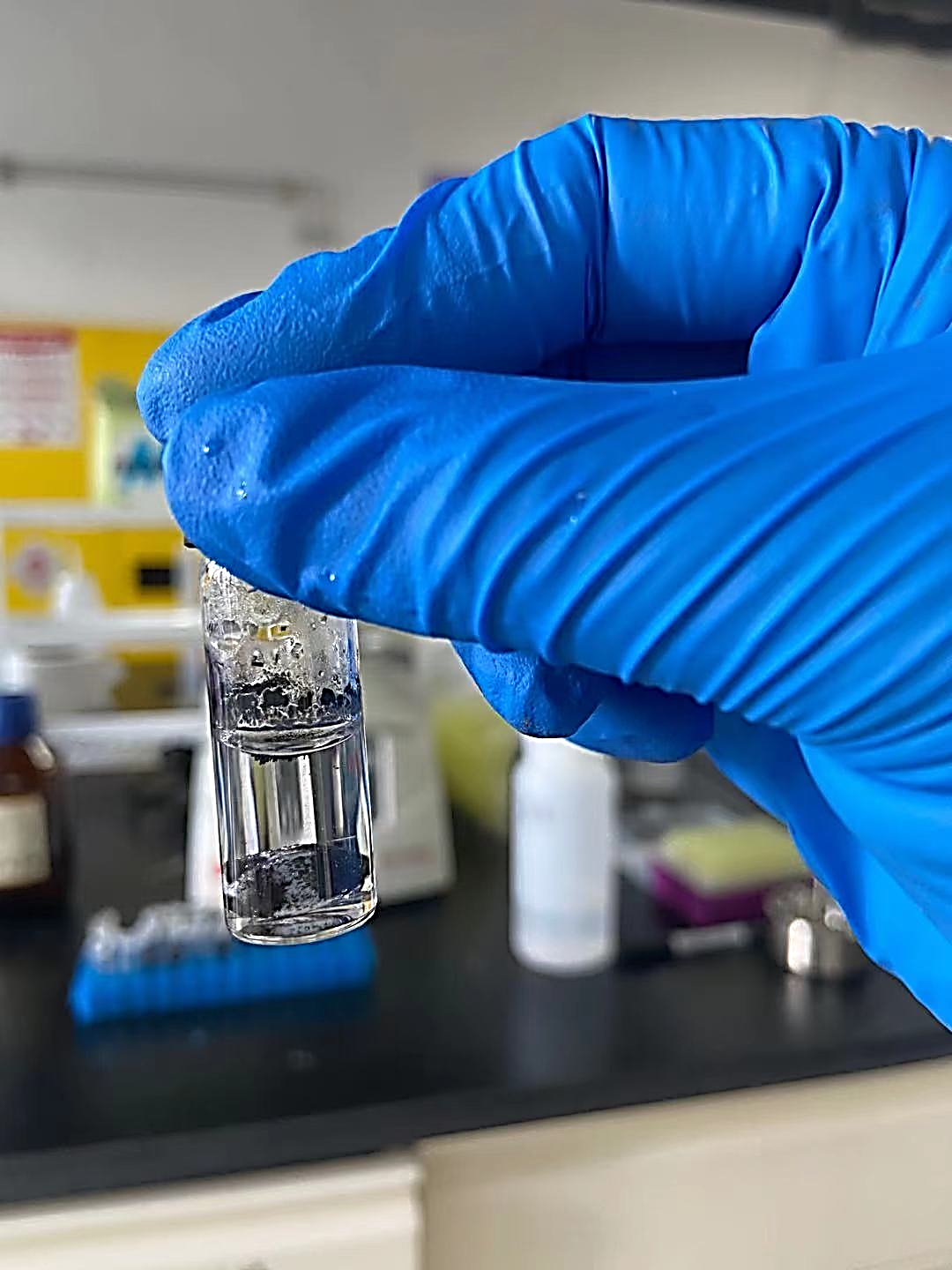


**Supplementary Figure S1.** The appearance of the treated CoBO_x_ catalysts.


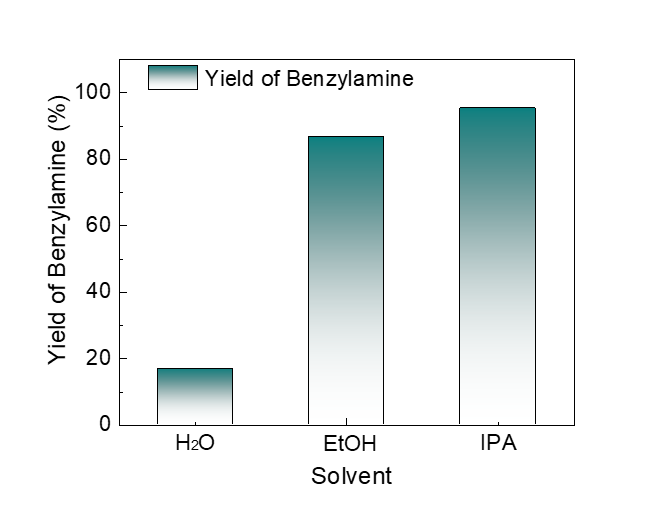


**Supplementary Figure S2.** Catalytic performance on different solvents.

**Reaction condition**: 0.5 mmol benzaldehyde, 2 mL ammonia, 80 ℃, 2 MPa H_2_, 20 mg CoBO_x_, 15 h


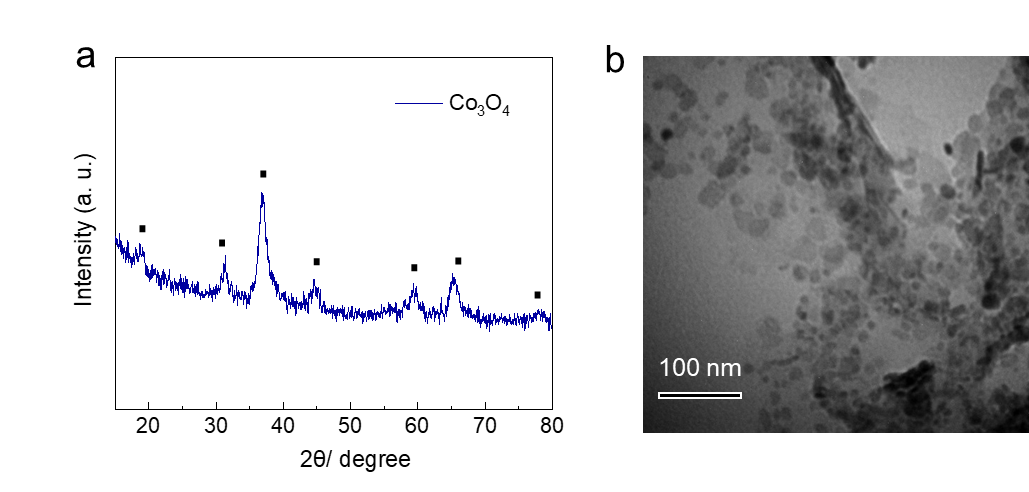


**Supplementary Figure S3.** (a) The XRD pattern and (b) TEM image of the synthesized Co_3_O_4_.


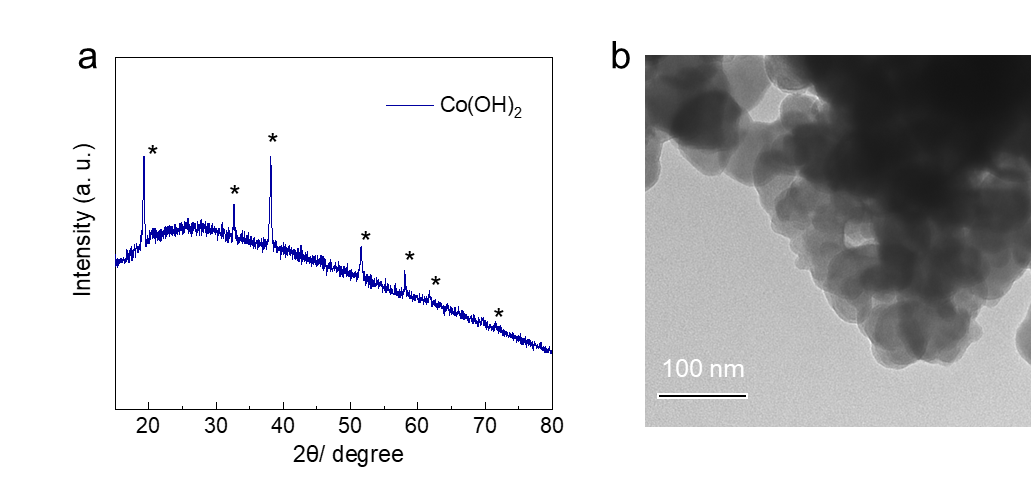


**Supplementary Figure S4.** (a) The XRD pattern and (b) TEM image of the synthesized Co(OH)_2_.

## Supplementary Tables

**Supplementary Table S1**. Comparison on different Co catalysts.

| **Co catalysts** | **Yield of benzylamine (%)** |
| --- | --- |
| Co_3_O_4_ | <1 |
| Co(OH)_2_ | <1 |
| Co/C | 40.0% |
| CoBO_x_ | 95.2% |

**Reaction conditions:** 20 mg catalysts, 80 ℃, 2 mL ammonia (25-28 *wt.*%), 4 mL isopropanol, 15 h.

**Supplementary Table S2** The treatment of CoBO_x_ under different H_2_ pressure and ammonia.

| Sample | H_2_ Pressure  (MPa) | Ammonia  (mL) | In-situ formation of Co Nanoparticles |
| --- | --- | --- | --- |
| 1 | 1 | 0.5 | No |
| 2 | 1 | 2 | No |
| 3 | 1 | 3 | No |
| 4 | 2 | 0.5 | No |
| 5 | 2 | 2 | Yes |
| 6 | 2 | 3 | Yes |

**Note**: 20 mg CoBO_x_ was treated under 80 ℃, 4 mL isopropanol, and 0.5 mmol benzaldehyde for 15 h.
